# A second polymorph of chlorido(hydroxy­diphenyl­phosphane)gold(I)

**DOI:** 10.1107/S1600536811035732

**Published:** 2011-09-14

**Authors:** Sicelo V. Sithole, Richard J. Staples, Werner E. Van Zyl

**Affiliations:** a School of Chemistry, University of KwaZulu-Natal, Westville Campus, Private, Bag X54001, Durban, 4000, South Africa; b Department of Chemistry, Michigan State University, East Lansing, MI, 48824-1322, USA

## Abstract

The title complex, [AuCl{(C_6_H_5_)_2_P(OH)-κ*P*}] or [AuCl(C_12_H_11_OP)], contains two independent mol­ecules in the asymmetric unit and is a polymorph of a previously reported structure [Hollatz *et al.* (1999 ▶) *J. Chem. Soc. Dalton Trans*. pp. 111–114]. The crystal structure exhibits inter­molecular Au⋯Au inter­actions with alternate distances of 3.0112 (3) Å and 3.0375 (2) Å. The Cl—Au—P bond angle varies between different mol­ecular units, depending on the degree of influence of the intra­molecular the O—H⋯Cl hydrogen bond; the angle thus varies between negligible distortion from linearity at 179.23 (3)° and more significant distortion at 170.39 (4)°, which differs from the previously reported polymorph in which both these angles are approximately 170°. The Au—Cl [2.3366 (9) and 2.3131 (10)Å] and Au—P [2.2304 (10) and 2.2254 (10) Å] bond lengths vary slightly between the two independent mol­ecules but overall, the bond lengths are in good agreement with those in the previously reported polymorph.

## Related literature

For background to polymorphism, see: Braga & Grepioni (2007) ▶. Polymorphs of chloro­gold(I) phosphine complexes are relatively common (Healy, 2003 ▶) and often display inter­esting photochemical properties (Hoshino *et al.*, 2010 ▶). For the previously reported polymorph of the title compound, see: Hollatz *et al.* (1999 ▶). For our studies on gold and P-based ligand complexes, see: Van Zyl (2010 ▶).
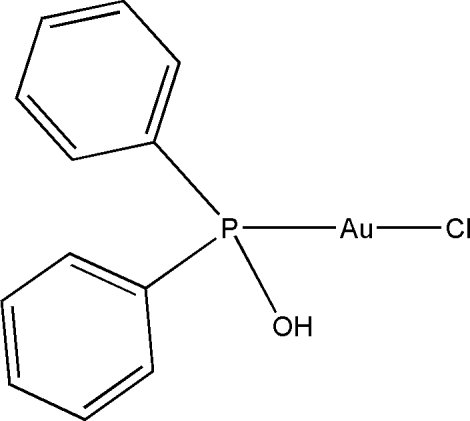


## Experimental

### 

#### Crystal data

[AuCl(C_12_H_11_OP)]
*M* *_r_* = 434.59
Monoclinic, 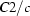

*a* = 29.2734 (18) Å
*b* = 10.2321 (6) Å
*c* = 17.5643 (11) Å
β = 106.483 (1)°
*V* = 5044.8 (5) Å^3^
*Z* = 16
Mo *K*α radiation
μ = 11.98 mm^−1^
*T* = 173 K
0.32 × 0.13 × 0.06 mm

#### Data collection

Bruker APEXII CCD diffractometer
Absorption correction: multi-scan (*SADABS*; Bruker, 2008 ▶) *T*_min_ = 0.371, *T*_max_ = 0.745
18295 measured reflections
4651 independent reflections
4183 reflections with *I* > 2σ(*I*)
*R*_int_ = 0.036

#### Refinement

*R*[*F* ^2^ > 2σ(*F* ^2^)] = 0.019
*wR*(*F* ^2^) = 0.048
*S* = 1.03
4651 reflections
291 parameters
H-atom parameters constrained
Δρ_max_ = 0.74 e Å^−3^
Δρ_min_ = −0.63 e Å^−3^

### 

Data collection: *APEX2* (Bruker, 2008 ▶); cell refinement: *SAINT* (Bruker, 2008 ▶); data reduction: *SAINT*; program(s) used to solve structure: *SHELXS97* (Sheldrick, 2008 ▶); program(s) used to refine structure: *SHELXL97* (Sheldrick, 2008 ▶); molecular graphics: *SHELXTL* (Sheldrick, 2008 ▶); software used to prepare material for publication: *SHELXTL*.

## Supplementary Material

Crystal structure: contains datablock(s) I, global. DOI: 10.1107/S1600536811035732/ru2011sup1.cif

Structure factors: contains datablock(s) I. DOI: 10.1107/S1600536811035732/ru2011Isup2.hkl

Additional supplementary materials:  crystallographic information; 3D view; checkCIF report

## Figures and Tables

**Table 1:** Hydrogen-bond geometry (Å, °)

*D*—H⋯*A*	*D*—H	H⋯*A*	*D*⋯*A*	*D*—H⋯*A*
O1—H1⋯Cl2	0.84	2.16	2.994 (3)	170
O2—H2⋯Cl1	0.84	2.23	3.050 (3)	166

**Fig. 1.**
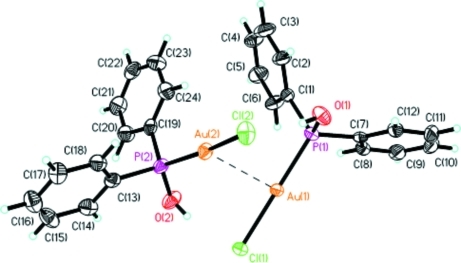
The molecular structure of (I), showing the intermolecular Au—Au interaction between two units with atom labels and 50% probability displacement ellipsoids for non-H atoms.

**Fig. 2.**
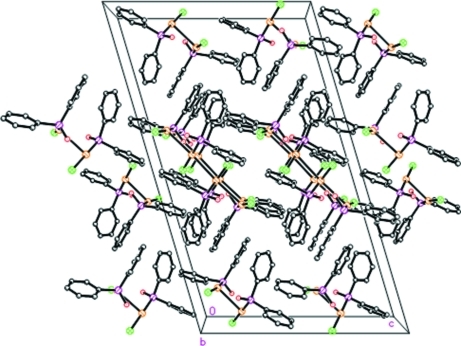
The packing of the crystal structure, viewed along the a axis.

**Fig. 3.**
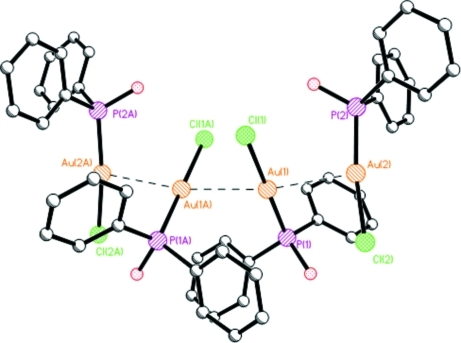
A molecular drawing showing four units, two inner and two outer. Note the atoms of the inner P—Au—Cl moiety points in approximately the same direction, whilst the outer two in the opposite direction.

## supplementary crystallographic information

### Comment

Polymorphism is generally described as the ability of the same chemical substance to exist in at least two different crystalline forms (Braga & Grepioni 2007). Data collection at 173 K showed that the gold(I) compound, (I), had crystallized in monoclinic space group *C*2/*c* with sixteen formula units per unit cell (final *R* value 0.019) which differs significantly from a previously reported crystal structure of this compound (Hollatz *et al.*, 1999), obtained at 195 K in triclinic space group P1 with four formula units per unit cell and a final *R* value of 0.036. Due to the nearness of the respective data collection temperatures, we disregard an interpretation of this result as indicating that the structure had undergone a significant phase transition between 173 and 195 K, and thus conclude that the structure of complex (I) presented here is a genuine polymorph and not the consequence of a phase transition. Indeed, polymorphs of chlorogold(I) phosphine complexes are relatively common (Healy, 2003) and often display interesting photochemical properties (Hoshino *et al.*, 2010).

In our continued studies on gold and P-based ligand complexes (Van Zyl, 2010), the title complex [AuCl{(C_6_H_5_)_2_P(OH)}], (I), was readily synthesized from the reaction between Ph_2_PCl in wet dichloromethane (*i.e.* containing traces of water) followed by addition of [AuCl(tht)] (tht = tetrahydrothiophene). In the previously reported study of the polymorph, [AuCl(Me_2_S)] was reacted with Ph_2_P(OH) in CH_2_Cl_2_ solvent with the elimination of Me_2_S, forming [AuCl{(C_6_H_5_)_2_P(OH)}]. A solution ^31^P NMR study showed a sharp singlet at δ = 89.5 for (I) which corresponds well with the value of δ = 90.4 for the polymorph (Hollatz *et al.*, 1999). Since polymorphs must have the same resonance in solution, and since the same solvent (CDCl_3_) was used in both cases, the small difference (0.9 p.p.m.) is ascribed to possible difference in temperature (293 *versus* 298 K) during data acquisition. A single-crystal X-ray analysis of the compound subsequently provided unambiguous proof of the authenticity of the complex, and for it to be a polymorph.

The crystal structure of (I) presented here includes four molecular units along a virtual chain (described as two "inner" and two "outer" units) all linked through intermolecular Au···Au interactions with alternate distances of 3.0112 (3) Å (between the two inner units) and 3.0375 (2) Å between an inner and outer unit which are both shorter than the corresponding distance for the reported polymorph, at 3.1112 (7) Å. The Cl—Au—P bond angles between the two inner complexes have in one case negligible distortion away from linearity at 179.23 (3)° while in the other case it has significant distortion at 170.39 (4)°, which differs from the previously reported polymorph where both these angles are approximately 170°. This difference originates through the varying influence of O—H···Cl type hydrogen bonding within the respective molecular units: the stronger the H-bonding, the more the distortion. In the case of (I), the one Cl—Au—P unit is positioned too far from a P—O—H unit for any O—H···Cl hydrogen bonding [*d*(H···Cl) = 2.23 Å] to occur whilst the other Cl—Au—P unit is much closer to a P—O—H unit at d(H···Cl) = 2.16 Å, and this causes the observed distortion. In the triclinic polymorph, hydrogen bonding is present on both monomeric units at d(H···Cl) = 2.03 and 2.11 Å, respectively, which leads to significant distortion from linearity for both Cl—Au—P units.The Au—Cl bond length of the inner unit is 2.3366 (9) and for the outer unit 2.3131 (10) Å, respectively, whilst the Au—P bond lengths are slightly shorter at 2.2304 (10) (inner) and 2.2254 (10) Å (outer), respectively; these bond length results are in good agreement with the previously reported structure. The P—O bond length in (I) is 1.592 (3) Å *versus* 1.597 (5) Å in the triclinic polymorph. Based on the current studies, it cannot readily be inferred whether the polymorph with the shorter Au···Au interactions is the thermodynamically more stable of the two. Note that structure (I) has a slightly lower calculated density at 2.289 g/cm^3^ compared to the other polymorph at 2.309 g/cm^3^, suggesting the molecular packing in the latter is more efficient, presumably resulting from a larger extent of hydrogen bonding.

### Experimental

Preparation and characterization of complex (I): A Schlenk flask equipped with a magnetic stirrer bar was charged with wet dichloromethane (5 ml) and this was followed by addition of ClPPh_2_ (0.210 ml, 1.11 mmol). The mixture was stirred for 20 minutes at room temperature. A dichloromethane solution of [AuCl(tht)] (354 mg, 1.11 mmol) was added in one portion and the resulting mixture stirred for a further 15 minutes. All of the solvent and tht were removed and the product isolated as a free-flowing white powder. ^31^P NMR (101 MHz, CDCl_3_, 298 K) *δ*_P_ = 89.2 (s, 1P). Single crystals were obtained by slow diffusion of hexane vapor into a saturated dichloromethane solution.

### Refinement

All H atoms were placed in calculated positions and refined using a riding model. C—H(aromatic) = 0.94 Å and *U*_iso_(H) = 1.2Ueq(C) C—H (alaphatic) = 0.99 Å and *U*_iso_(H) = 1.2Ueq(C) CH2 = 0.98 Å and *U*_iso_(H) = 1.2Ueq(C) CH3 = 0.97Å and *U*_iso_(H) = 1.5Ueq(C) N—H = 0.86 (0.92)Å and *U*_iso_(H) = 1.2 *U*_eq_(N) O—H(alcohol) = 0.85Åand *U*_iso_(H) = 1.2Ueq(O) O—H(acid) = 0.82 Å and *U*_iso_(H) = 1.5Ueq(O).

### Crystal data

**Table:**

[AuCl(C_12_H_11_OP)]	*F*(000) = 3232
*M**_r_* = 434.59	*D*_x_ = 2.289 Mg m^−^^3^
Monoclinic, *C*2/*c*	Mo *K*α radiation, λ = 0.71073 Å
Hall symbol: -C 2yc	Cell parameters from 9950 reflections
*a* = 29.2734 (18) Å	θ = 2.3–25.4°
*b* = 10.2321 (6) Å	µ = 11.98 mm^−^^1^
*c* = 17.5643 (11) Å	*T* = 173 K
β = 106.483 (1)°	Chunk, colourless
*V* = 5044.8 (5) Å^3^	0.32 × 0.13 × 0.06 mm
*Z* = 16	

### Data collection

**Table:**

Bruker APEXII CCD diffractometer	4651 independent reflections
Radiation source: fine-focus sealed tube	4183 reflections with *I* > 2σ(*I*)
graphite	*R*_int_ = 0.036
Detector resolution: 836.6 pixels mm^-1^	θ_max_ = 25.4°, θ_min_ = 2.1°
ω,and/f 0.5 deg scans	*h* = −35→35
Absorption correction: multi-scan (*SADABS*; Bruker, 2008)	*k* = −12→12
*T*_min_ = 0.371, *T*_max_ = 0.745	*l* = −21→21
18295 measured reflections	

### Refinement

**Table:**

Refinement on *F*^2^	Primary atom site location: structure-invariant direct methods
Least-squares matrix: full	Secondary atom site location: difference Fourier map
*R*[*F*^2^ > 2σ(*F*^2^)] = 0.019	Hydrogen site location: inferred from neighbouring sites
*wR*(*F*^2^) = 0.048	H-atom parameters constrained
*S* = 1.03	*w* = 1/[σ^2^(*F*_o_^2^) + (0.0223*P*)^2^] where *P* = (*F*_o_^2^ + 2*F*_c_^2^)/3
4651 reflections	(Δ/σ)_max_ = 0.004
291 parameters	Δρ_max_ = 0.74 e Å^−^^3^
0 restraints	Δρ_min_ = −0.63 e Å^−^^3^

### Special details

**Table:**

Experimental. Data was collected using a BRUKER CCD (charge coupled device) based diffractometer equipped with an Oxford low-temperature apparatus operating at 173 K. A suitable crystal was chosen and mounted on a glass fiber or nylon loop using Paratone oil for Mo radiation and Mineral oil for Copper radiation. Data were measured using omega and phi scans of 0.5° per frame for 30 s. The total number of images were based on results from the program COSMO where redundancy was expected to be 4 and completeness to 0.83Å to 100%. Cell parameters were retrieved using *APEX* II software and refined using *SAINT* on all observed reflections.Data reduction was performed using the *SAINT* software which corrects for Lp. Scaling and absorption corrections were applied using *SADABS6* multi-scan technique, supplied by George Sheldrick. The structures are solved by the direct method using the *SHELXS97* program and refined by least squares method on F2, *SHELXL97*, incorporated in *SHELXTL*-PC V 6.14. The crystal used for the diffraction study showed no decomposition during data collection.
Geometry. All e.s.d.'s (except the e.s.d. in the dihedral angle between two l.s. planes) are estimated using the full covariance matrix. The cell e.s.d.'s are taken into account individually in the estimation of e.s.d.'s in distances, angles and torsion angles; correlations between e.s.d.'s in cell parameters are only used when they are defined by crystal symmetry. An approximate (isotropic) treatment of cell e.s.d.'s is used for estimating e.s.d.'s involving l.s. planes.
Refinement. Refinement of *F*^2^ against ALL reflections. The weighted *R*-factor *wR* and goodness of fit *S* are based on *F*^2^, conventional *R*-factors *R* are based on *F*, with *F* set to zero for negative *F*^2^. The threshold expression of *F*^2^ > σ(*F*^2^) is used only for calculating *R*-factors(gt) *etc*. and is not relevant to the choice of reflections for refinement. *R*-factors based on *F*^2^ are statistically about twice as large as those based on *F*, and *R*- factors based on ALL data will be even larger.

### Fractional atomic coordinates and isotropic or equivalent isotropic displacement parameters (Å^2^)

**Table:**

	*x*	*y*	*z*	*U*_iso_*/*U*_eq_	
Au1	0.960725 (5)	0.437105 (13)	0.289805 (8)	0.02121 (5)	
Au2	0.887378 (5)	0.384820 (13)	0.378563 (9)	0.02700 (6)	
Cl1	1.00671 (3)	0.26698 (9)	0.36128 (6)	0.0284 (2)	
Cl2	0.89312 (4)	0.59742 (9)	0.42511 (6)	0.0368 (3)	
P1	0.91703 (4)	0.60110 (9)	0.22307 (6)	0.0228 (2)	
P2	0.87692 (4)	0.17285 (9)	0.35069 (7)	0.0268 (2)	
O1	0.90588 (11)	0.7154 (2)	0.27649 (16)	0.0342 (7)	
H1	0.9059	0.6853	0.3210	0.051*	
O2	0.91734 (10)	0.0976 (3)	0.3237 (2)	0.0411 (8)	
H2	0.9435	0.1363	0.3419	0.062*	
C1	0.85968 (13)	0.5459 (4)	0.1619 (2)	0.0241 (8)	
C2	0.81825 (15)	0.6080 (4)	0.1658 (3)	0.0372 (11)	
H2B	0.8198	0.6785	0.2016	0.045*	
C3	0.77481 (17)	0.5670 (5)	0.1175 (3)	0.0508 (13)	
H3	0.7465	0.6095	0.1201	0.061*	
C4	0.77226 (16)	0.4651 (5)	0.0657 (3)	0.0471 (12)	
H4	0.7423	0.4389	0.0316	0.056*	
C5	0.81274 (17)	0.4011 (5)	0.0630 (3)	0.0437 (11)	
H5	0.8107	0.3294	0.0278	0.052*	
C6	0.85684 (15)	0.4403 (4)	0.1113 (3)	0.0347 (10)	
H6	0.8849	0.3951	0.1096	0.042*	
C7	0.94390 (13)	0.6894 (3)	0.1576 (2)	0.0244 (8)	
C8	0.95612 (14)	0.6238 (4)	0.0969 (2)	0.0286 (9)	
H8	0.9491	0.5334	0.0889	0.034*	
C9	0.97805 (15)	0.6877 (4)	0.0485 (3)	0.0378 (10)	
H9	0.9862	0.6416	0.0073	0.045*	
C10	0.98827 (15)	0.8194 (5)	0.0597 (3)	0.0428 (12)	
H10	1.0038	0.8638	0.0265	0.051*	
C11	0.97600 (16)	0.8859 (4)	0.1187 (3)	0.0434 (12)	
H11	0.9827	0.9767	0.1256	0.052*	
C12	0.95387 (14)	0.8222 (4)	0.1687 (3)	0.0330 (10)	
H12	0.9457	0.8687	0.2097	0.040*	
C13	0.87130 (14)	0.0853 (4)	0.4367 (2)	0.0269 (9)	
C14	0.88671 (15)	−0.0425 (4)	0.4518 (3)	0.0345 (10)	
H14	0.9010	−0.0863	0.4167	0.041*	
C15	0.88123 (17)	−0.1070 (4)	0.5187 (3)	0.0425 (12)	
H15	0.8916	−0.1950	0.5287	0.051*	
C16	0.86084 (17)	−0.0442 (4)	0.5704 (3)	0.0405 (11)	
H16	0.8574	−0.0881	0.6161	0.049*	
C17	0.84557 (16)	0.0823 (4)	0.5551 (3)	0.0368 (10)	
H17	0.8311	0.1253	0.5902	0.044*	
C18	0.85078 (14)	0.1480 (4)	0.4900 (2)	0.0298 (9)	
H18	0.8405	0.2361	0.4810	0.036*	
C19	0.82412 (14)	0.1317 (4)	0.2729 (2)	0.0273 (9)	
C20	0.81456 (16)	0.0011 (4)	0.2501 (3)	0.0361 (10)	
H20	0.8365	−0.0655	0.2744	0.043*	
C21	0.77345 (16)	−0.0307 (4)	0.1926 (3)	0.0407 (11)	
H21	0.7673	−0.1193	0.1769	0.049*	
C22	0.74071 (16)	0.0648 (4)	0.1570 (3)	0.0393 (11)	
H22	0.7120	0.0419	0.1180	0.047*	
C23	0.75038 (16)	0.1929 (4)	0.1789 (3)	0.0430 (11)	
H23	0.7283	0.2592	0.1549	0.052*	
C24	0.79207 (16)	0.2261 (4)	0.2357 (3)	0.0376 (10)	
H24	0.7987	0.3154	0.2493	0.045*	

### Atomic displacement parameters (Å^2^)

**Table:**

	*U*^11^	*U*^22^	*U*^33^	*U*^12^	*U*^13^	*U*^23^
Au1	0.01960 (9)	0.02248 (8)	0.02120 (9)	0.00212 (5)	0.00521 (7)	0.00271 (5)
Au2	0.02700 (10)	0.02272 (9)	0.03339 (10)	−0.00234 (6)	0.01200 (7)	−0.00352 (6)
Cl1	0.0240 (5)	0.0277 (5)	0.0322 (5)	0.0054 (4)	0.0060 (4)	0.0078 (4)
Cl2	0.0522 (7)	0.0266 (5)	0.0372 (6)	−0.0047 (5)	0.0217 (5)	−0.0079 (4)
P1	0.0238 (6)	0.0214 (5)	0.0219 (5)	0.0035 (4)	0.0042 (4)	0.0016 (4)
P2	0.0232 (6)	0.0237 (5)	0.0351 (6)	−0.0008 (4)	0.0106 (5)	−0.0041 (4)
O1	0.0470 (19)	0.0264 (14)	0.0300 (17)	0.0066 (13)	0.0120 (15)	0.0004 (12)
O2	0.0306 (18)	0.0311 (15)	0.067 (2)	−0.0012 (13)	0.0237 (17)	−0.0126 (15)
C1	0.021 (2)	0.029 (2)	0.021 (2)	0.0005 (16)	0.0051 (17)	0.0061 (16)
C2	0.023 (2)	0.038 (2)	0.050 (3)	0.0052 (18)	0.011 (2)	−0.002 (2)
C3	0.023 (3)	0.057 (3)	0.072 (4)	0.009 (2)	0.014 (3)	0.000 (3)
C4	0.023 (3)	0.061 (3)	0.049 (3)	−0.004 (2)	−0.004 (2)	0.009 (2)
C5	0.035 (3)	0.053 (3)	0.037 (3)	−0.004 (2)	0.000 (2)	−0.005 (2)
C6	0.023 (2)	0.042 (2)	0.039 (3)	0.0021 (18)	0.008 (2)	−0.0097 (19)
C7	0.017 (2)	0.0268 (19)	0.024 (2)	0.0019 (15)	−0.0030 (16)	0.0059 (16)
C8	0.026 (2)	0.031 (2)	0.028 (2)	0.0032 (17)	0.0051 (18)	0.0061 (17)
C9	0.033 (3)	0.052 (3)	0.026 (2)	0.001 (2)	0.0030 (19)	0.013 (2)
C10	0.025 (3)	0.057 (3)	0.043 (3)	−0.007 (2)	0.004 (2)	0.021 (2)
C11	0.033 (3)	0.035 (2)	0.052 (3)	−0.013 (2)	−0.005 (2)	0.009 (2)
C12	0.027 (2)	0.028 (2)	0.038 (3)	−0.0015 (17)	−0.0002 (19)	0.0044 (18)
C13	0.021 (2)	0.0249 (19)	0.030 (2)	−0.0050 (16)	0.0000 (17)	−0.0040 (17)
C14	0.032 (3)	0.031 (2)	0.036 (3)	0.0003 (18)	0.003 (2)	−0.0067 (18)
C15	0.053 (3)	0.025 (2)	0.040 (3)	−0.002 (2)	−0.002 (2)	0.0035 (19)
C16	0.052 (3)	0.035 (2)	0.029 (3)	−0.010 (2)	0.003 (2)	0.0008 (19)
C17	0.044 (3)	0.035 (2)	0.032 (3)	−0.006 (2)	0.012 (2)	−0.0046 (19)
C18	0.029 (2)	0.0255 (19)	0.033 (2)	−0.0002 (17)	0.0047 (19)	−0.0011 (17)
C19	0.031 (2)	0.028 (2)	0.028 (2)	−0.0024 (17)	0.0159 (19)	−0.0002 (16)
C20	0.036 (3)	0.028 (2)	0.041 (3)	−0.0002 (19)	0.007 (2)	−0.0019 (19)
C21	0.043 (3)	0.039 (2)	0.040 (3)	−0.009 (2)	0.012 (2)	−0.010 (2)
C22	0.029 (3)	0.059 (3)	0.029 (2)	−0.012 (2)	0.006 (2)	−0.002 (2)
C23	0.035 (3)	0.048 (3)	0.041 (3)	0.007 (2)	0.003 (2)	0.007 (2)
C24	0.040 (3)	0.031 (2)	0.042 (3)	0.0003 (19)	0.011 (2)	0.0010 (19)

### Geometric parameters (Å, °)

**Table:**

Au1—P1	2.2304 (10)	C9—C10	1.382 (6)
Au1—Cl1	2.3366 (9)	C9—H9	0.9500
Au1—Au1^i^	3.0112 (3)	C10—C11	1.370 (7)
Au1—Au2	3.0375 (2)	C10—H10	0.9500
Au2—P2	2.2254 (10)	C11—C12	1.392 (6)
Au2—Cl2	2.3131 (10)	C11—H11	0.9500
P1—O1	1.591 (3)	C12—H12	0.9500
P1—C1	1.808 (4)	C13—C14	1.385 (5)
P1—C7	1.808 (4)	C13—C18	1.403 (5)
P2—O2	1.592 (3)	C14—C15	1.396 (6)
P2—C19	1.799 (4)	C14—H14	0.9500
P2—C13	1.803 (4)	C15—C16	1.379 (6)
O1—H1	0.8400	C15—H15	0.9500
O2—H2	0.8400	C16—C17	1.371 (6)
C1—C2	1.387 (5)	C16—H16	0.9500
C1—C6	1.387 (5)	C17—C18	1.371 (6)
C2—C3	1.378 (6)	C17—H17	0.9500
C2—H2B	0.9500	C18—H18	0.9500
C3—C4	1.372 (7)	C19—C24	1.375 (6)
C3—H3	0.9500	C19—C20	1.401 (5)
C4—C5	1.366 (6)	C20—C21	1.373 (6)
C4—H4	0.9500	C20—H20	0.9500
C5—C6	1.387 (6)	C21—C22	1.387 (6)
C5—H5	0.9500	C21—H21	0.9500
C6—H6	0.9500	C22—C23	1.373 (6)
C7—C8	1.389 (5)	C22—H22	0.9500
C7—C12	1.392 (5)	C23—C24	1.381 (6)
C8—C9	1.368 (5)	C23—H23	0.9500
C8—H8	0.9500	C24—H24	0.9500
			
P1—Au1—Cl1	179.23 (3)	C8—C9—H9	120.1
P1—Au1—Au1^i^	98.95 (3)	C10—C9—H9	120.1
Cl1—Au1—Au1^i^	81.37 (2)	C11—C10—C9	119.9 (4)
P1—Au1—Au2	91.08 (3)	C11—C10—H10	120.0
Cl1—Au1—Au2	88.65 (2)	C9—C10—H10	120.0
Au1^i^—Au1—Au2	169.256 (4)	C10—C11—C12	120.9 (4)
P2—Au2—Cl2	170.39 (4)	C10—C11—H11	119.5
P2—Au2—Au1	97.58 (3)	C12—C11—H11	119.5
Cl2—Au2—Au1	91.41 (3)	C11—C12—C7	119.1 (4)
O1—P1—C1	105.59 (17)	C11—C12—H12	120.5
O1—P1—C7	101.93 (16)	C7—C12—H12	120.5
C1—P1—C7	106.06 (17)	C14—C13—C18	118.8 (4)
O1—P1—Au1	115.24 (11)	C14—C13—P2	121.9 (3)
C1—P1—Au1	112.00 (12)	C18—C13—P2	119.4 (3)
C7—P1—Au1	114.96 (12)	C13—C14—C15	119.9 (4)
O2—P2—C19	102.24 (18)	C13—C14—H14	120.0
O2—P2—C13	105.13 (18)	C15—C14—H14	120.0
C19—P2—C13	104.90 (17)	C16—C15—C14	120.5 (4)
O2—P2—Au2	117.99 (11)	C16—C15—H15	119.7
C19—P2—Au2	115.47 (13)	C14—C15—H15	119.7
C13—P2—Au2	109.84 (13)	C17—C16—C15	119.3 (4)
P1—O1—H1	109.5	C17—C16—H16	120.4
P2—O2—H2	109.5	C15—C16—H16	120.4
C2—C1—C6	119.6 (4)	C16—C17—C18	121.3 (4)
C2—C1—P1	120.4 (3)	C16—C17—H17	119.4
C6—C1—P1	120.1 (3)	C18—C17—H17	119.4
C3—C2—C1	119.8 (4)	C17—C18—C13	120.2 (4)
C3—C2—H2B	120.1	C17—C18—H18	119.9
C1—C2—H2B	120.1	C13—C18—H18	119.9
C4—C3—C2	120.4 (4)	C24—C19—C20	118.7 (4)
C4—C3—H3	119.8	C24—C19—P2	121.3 (3)
C2—C3—H3	119.8	C20—C19—P2	120.0 (3)
C5—C4—C3	120.2 (4)	C21—C20—C19	119.9 (4)
C5—C4—H4	119.9	C21—C20—H20	120.1
C3—C4—H4	119.9	C19—C20—H20	120.1
C4—C5—C6	120.4 (4)	C20—C21—C22	121.0 (4)
C4—C5—H5	119.8	C20—C21—H21	119.5
C6—C5—H5	119.8	C22—C21—H21	119.5
C1—C6—C5	119.6 (4)	C23—C22—C21	119.0 (4)
C1—C6—H6	120.2	C23—C22—H22	120.5
C5—C6—H6	120.2	C21—C22—H22	120.5
C8—C7—C12	119.3 (4)	C22—C23—C24	120.5 (4)
C8—C7—P1	120.0 (3)	C22—C23—H23	119.8
C12—C7—P1	120.7 (3)	C24—C23—H23	119.8
C9—C8—C7	121.0 (4)	C19—C24—C23	120.9 (4)
C9—C8—H8	119.5	C19—C24—H24	119.5
C7—C8—H8	119.5	C23—C24—H24	119.5
C8—C9—C10	119.9 (4)		
			
P1—Au1—Au2—P2	126.72 (4)	C1—P1—C7—C12	116.5 (3)
Cl1—Au1—Au2—P2	−54.00 (4)	Au1—P1—C7—C12	−119.1 (3)
Au1^i^—Au1—Au2—P2	−32.28 (5)	C12—C7—C8—C9	0.5 (6)
P1—Au1—Au2—Cl2	−56.69 (4)	P1—C7—C8—C9	−177.6 (3)
Cl1—Au1—Au2—Cl2	122.59 (4)	C7—C8—C9—C10	−0.1 (6)
Au1^i^—Au1—Au2—Cl2	144.31 (5)	C8—C9—C10—C11	−0.6 (7)
Cl1—Au1—P1—O1	−11 (3)	C9—C10—C11—C12	0.9 (7)
Au1^i^—Au1—P1—O1	−125.53 (13)	C10—C11—C12—C7	−0.5 (6)
Au2—Au1—P1—O1	58.35 (13)	C8—C7—C12—C11	−0.2 (6)
Cl1—Au1—P1—C1	−132 (3)	P1—C7—C12—C11	177.9 (3)
Au1^i^—Au1—P1—C1	113.74 (13)	O2—P2—C13—C14	−20.8 (4)
Au2—Au1—P1—C1	−62.38 (14)	C19—P2—C13—C14	86.6 (4)
Cl1—Au1—P1—C7	107 (3)	Au2—P2—C13—C14	−148.7 (3)
Au1^i^—Au1—P1—C7	−7.40 (14)	O2—P2—C13—C18	159.2 (3)
Au2—Au1—P1—C7	176.48 (14)	C19—P2—C13—C18	−93.4 (3)
Cl2—Au2—P2—O2	−139.1 (3)	Au2—P2—C13—C18	31.3 (3)
Au1—Au2—P2—O2	20.00 (16)	C18—C13—C14—C15	0.6 (6)
Cl2—Au2—P2—C19	99.6 (3)	P2—C13—C14—C15	−179.4 (3)
Au1—Au2—P2—C19	−101.26 (14)	C13—C14—C15—C16	−0.4 (7)
Cl2—Au2—P2—C13	−18.8 (3)	C14—C15—C16—C17	0.6 (7)
Au1—Au2—P2—C13	140.40 (14)	C15—C16—C17—C18	−0.9 (7)
O1—P1—C1—C2	3.3 (4)	C16—C17—C18—C13	1.1 (6)
C7—P1—C1—C2	−104.4 (3)	C14—C13—C18—C17	−1.0 (6)
Au1—P1—C1—C2	129.4 (3)	P2—C13—C18—C17	179.0 (3)
O1—P1—C1—C6	−176.2 (3)	O2—P2—C19—C24	−131.4 (3)
C7—P1—C1—C6	76.1 (4)	C13—P2—C19—C24	119.0 (3)
Au1—P1—C1—C6	−50.0 (3)	Au2—P2—C19—C24	−2.0 (4)
C6—C1—C2—C3	−2.0 (6)	O2—P2—C19—C20	49.7 (3)
P1—C1—C2—C3	178.5 (4)	C13—P2—C19—C20	−59.8 (4)
C1—C2—C3—C4	0.0 (7)	Au2—P2—C19—C20	179.2 (3)
C2—C3—C4—C5	1.7 (8)	C24—C19—C20—C21	−1.1 (6)
C3—C4—C5—C6	−1.4 (7)	P2—C19—C20—C21	177.8 (3)
C2—C1—C6—C5	2.4 (6)	C19—C20—C21—C22	−0.7 (7)
P1—C1—C6—C5	−178.2 (3)	C20—C21—C22—C23	1.3 (7)
C4—C5—C6—C1	−0.7 (7)	C21—C22—C23—C24	−0.1 (7)
O1—P1—C7—C8	−175.6 (3)	C20—C19—C24—C23	2.3 (6)
C1—P1—C7—C8	−65.4 (3)	P2—C19—C24—C23	−176.6 (3)
Au1—P1—C7—C8	59.0 (3)	C22—C23—C24—C19	−1.7 (7)
O1—P1—C7—C12	6.3 (4)		

Symmetry codes: (i) −*x*+2, *y*, −*z*+1/2.

### Hydrogen-bond geometry (Å, °)

**Table:**

*D*—H···*A*	*D*—H	H···*A*	*D*···*A*	*D*—H···*A*
O1—H1···Cl2	0.84	2.16	2.994 (3)	170.
O2—H2···Cl1	0.84	2.23	3.050 (3)	166.
